# Tumour-promoting role of SOCS1 in colorectal cancer cells

**DOI:** 10.1038/srep14301

**Published:** 2015-09-22

**Authors:** William S. Tobelaim, Claudia Beaurivage, Audrey Champagne, Véronique Pomerleau, Aline Simoneau, Walid Chababi, Mehdi Yeganeh, Philippe Thibault, Roscoe Klinck, Julie C. Carrier, Gerardo Ferbeyre, Subburaj Ilangumaran, Caroline Saucier

**Affiliations:** 1 Department of Anatomy and Cell Biology, Faculty of Medicine and Health Sciences, Université de Sherbrooke, Sherbrooke, Quebec, J1E 4K8, Canada; 2 Department of Pediatrics and Immunology division, Faculty of Medicine and Health Sciences, Université de Sherbrooke, Sherbrooke, Quebec, J1E 4K8, Canada; 3 Department of Microbiology and Infectiology, Faculty of Medicine and Health Sciences, Université de Sherbrooke, Sherbrooke, Quebec, J1E 4K8, Canada; 4 Department of Biochemistry, Université de Montréal, Montréal, Quebec, H3C 3J7, Canada

## Abstract

The SOCS1 (Suppressor Of Cytokine Signalling 1) protein is considered a tumour suppressor. Notably, the *SOCS1* gene is frequently silenced in cancer by hypermethylation of its promoter. Besides blocking inflammation, SOCS1 tumour suppressor activity involves Met receptor inhibition and enhancement of p53 tumour suppressor activity. However, the role of SOCS1 in colorectal cancer (CRC) remains understudied and controversial. Here, we investigated SOCS1 relevance for CRC by querying gene expression datasets of human CRC specimens from The Cancer Genome Atlas (TCGA), and by SOCS1 gain/loss-of-function analyses in murine and human colon carcinoma cells. Our results show that *SOCS1* mRNA levels in tumours were more often elevated than reduced with respect to matched adjacent normal tissue of CRC specimens (n = 41). The analysis of TCGA dataset of 431 CRC patients revealed no correlation between *SOCS1* expression and overall survival. Overexpression of SOCS1 in CRC cells triggered cell growth enhancement, anchorage-independent growth and resistance to death stimuli, whereas knockdown of SOCS1 reduced these oncogenic features. Moreover, SOCS1 overexpression in mouse CT26 cells increased tumourigenesis *in vivo*. Biochemical analyses showed that SOCS1 pro-oncogenic activity correlated with the down-modulation of STAT1 expression. Collectively, these results suggest that SOCS1 may work as an oncogene in CRC.

The protein SOCS1 was first uncovered as a negative-feed back regulator of cytokine receptors that signal via the Janus family of tyrosine kinases (JAK) and the signal transducers and activators of transcription (STAT) proteins. Since then, SOCS1 is mainly recognized for its tumour-suppressing role^1^,^2^,^3^,^4^,^5^,^6^. Consistent with this idea, the gene encoding SOCS1 is frequently silenced in many cancers, such as by hypermethylation of its promoter in >50% hepatocellular carcinomas (HCC), pancreatic cancers, acute myeloid leukaemia and multiple myeloma^2^,^7^,^8^,^9^. In addition to blocking pro-inflammatory cytokine signalling, the tumour suppressor activity of SOCS1 has been linked to inhibition of hepatocyte growth factor (HGF)/Met receptor signalling and functions in hepatocytes^5^,^10^,^11^. Moreover, SOCS1 increases p53 phosphorylation (Ser15), DNA binding and transcriptional activity by forming a ternary complex with the DNA damage-regulated kinases ATM or ATR, which is critical for p53-dependent activation of genes involved in DNA repair, senescence and apoptosis^12^. These findings provide compelling evidence that SOCS1 works as a dominant tumour suppressor in HCC and underlie the molecular mechanisms involved.

In sharp contrast, the role of SOCS1 in other types of cancer, including CRC, is understudied and conflicting. For example, increased SOCS1 expression have been reported in human melanoma, neuroendocrine, breast and epidermal cancers^2^,^13^,^14^. Inconsistent with its alleged dominant tumour suppressor role, SOCS1 expression has been linked with tumour invasion and disease stage in melanoma^15^,^16^. Likewise conflicting observations with respect to its expression and function have emerged amongst the limited experimental and clinical studies that have investigated SOCS1 relevance in CRC. For instance, *SOCS1* gene methylation is rather uncommon in sporadic CRCs, ranging between 8–15% of the cases^17^,^18^. Nonetheless, methylation of the *SOCS1* promoter gene, together with that of the CpG island loci of other tumour suppressor genes, is a marker of a subset of CRCs referred to as the CpG island methylator phenotype (CIMP)^17^,^18^,^19^. Notably, CIMP colorectal tumours are associated with specific genetic features and poor clinical outcomes^20^,^21^, but *SOCS1* methylation in CIMP CRCs has been linked to better overall patient survival than those without^18^. Only two recently published studies have so far probed the abundance of *SOCS1* mRNA or protein in relatively small cohorts of human CRC samples^22^,^23^. Their findings are somewhat contradictory. In the David *et al.* study, the highest *SOCS1* mRNA and protein levels were seen in normal colon and early-stage adenomas, whereas the lowest levels were detected in advanced and poorly differentiated carcinomas^22^. Nonetheless, high SOCS1 protein level was still noted in 63% of advanced stage IV CRC tumours. Likewise, Ayyildiz *et al.* observed positive expression of SOCS1 in CRC tissues in nearly half of the cases by immunohistological analysis, but no association between SOCS1 protein level and clinicopathologic tumour characteristics^23^. Conflicting with a dominant tumour suppressor role for SOCS1 in CRC, elevated SOCS1 protein levels in CRC tumours did not predict better patient survival^23^.

Functional relevance of SOCS1 in CRC cells remains unresolved. Mouse studies indicate that SOCS1 influences CRC progression in a cell lineage-dependent manner. While mice with *Socs1* deletion in all tissues, except T and B cells, spontaneously developed colon inflammation and tumours^24^, its silencing in antigen-presenting macrophages and dendritic cells fostered anti-tumour immunity^25^,^26^. The role of SOCS1 in CRC cells has so far been investigated in a single published study by David *et al.*^22^. The authors concluded that SOCS1 works as a suppressor of metastasis, on the basis that its overexpression in human SW620 CRC cells reduced morphological transformation, invasion and metastasis without affecting proliferation, anchorage-independent growth or tumourigenesis^22^. However, an obvious increase in size of the colonies formed by SOCS1-expressing SW620 cells compared to control cells was not accounted for.

Overall, these limited numbers of studies have not yet settled whether or not SOCS1 is working as a tumour suppressor or as an oncogene in CRC. In this study, we have queried the clinical relevance of SOCS1 in human CRC patients by analysing gene expression datasets from The Cancer Genome Atlas (TCGA)^27^. Furthermore, SOCS1-regulated functions were investigated by gain- and loss-of-function studies in murine and human colon carcinoma cell models. Our results show a predominant up-regulation of *SOCS1* expression in human CRC tumours, but which did not correlate with better patient survival. Notably, we provide the first experimental evidence, both *in vitro* and *in vivo*, that SOCS1 fosters pro-oncogenic features in CRC cells.

## Results

### *SOCS1* mRNA expression is up-regulated in human CRC patient tumour specimens

**Figure 1:**
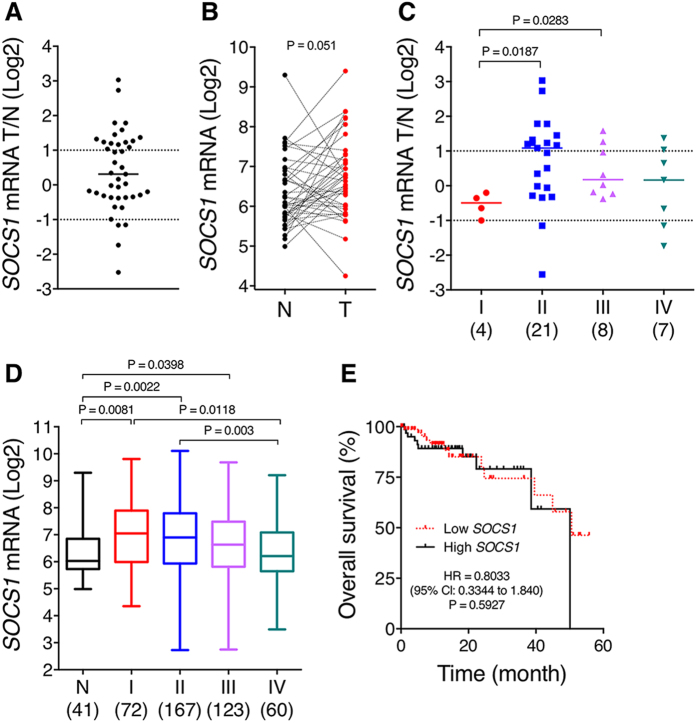
*SOCS1* is overexpressed in stage II CRC but its expression level does not correlate with overall survival. (**A**–**C**) *SOCS1* mRNA levels in 41-paired tumour and non-tumour margin tissue specimens of CRC patients obtained from TCGA gene expression profiling datasets are shown. *SOCS1* mRNA levels are expressed as tumour/non-tumour ratio (**A**), individually in tumour vs. non-tumour for each patient (**B**) and as a tumour/non-tumour ratio in each CRC stage (**C**). *SOCS1* is significantly overexpressed only in stage II CRC specimens based on Wilcoxon Signed Rank Test, P = 0.0216. (**D**) Graph shows *SOCS1* expression based on TCGA gene expression profiling datasets of 431 human CRC patients in human non-tumour colon vs. CRC specimens stratified according to stages. Number of tissues in each group is shown in brackets. (**E**) Kaplan-Meier overall survival curves according to *SOCS1* mRNA level in CRC patients (lower vs upper quartiles). *SOCS1* expression in CRC tumour does not correlate with overall survival (Hazard ratio of low/high of *SOCS1* = 0.8033).

The value of *SOCS1* expression as a predictor of human CRC progression has not been extensively explored. This prompted us to analyse *SOCS1* gene expression in human CRC based on publically available TCGA HiSeq RNA sequencing (RNA-Seq) gene expression profiling datasets of human CRC samples^27^. At first, *SOCS1* mRNA expression between tumour and matched normal tissue specimens of 41 patients included in TCGA gene expression datasets was evaluated. As shown in Fig. 1A, *SOCS1* gene expression levels were more often overexpressed than under-expressed in CRC tumours relative to non-tumour tissues. While 15 (37%) of the 41 CRC patients exhibited above 2-fold elevation of *SOCS1* mRNA in tumours, only 4 individuals (10%) showed below 2-fold under-expression of *SOCS1* in tumours. However, there was no significant difference in *SOCS1* mRNA expression between normal and tumour tissues based on a Wilcoxon matched-pairs signed rank test (Fig. 1B, Median difference in *SOCS1* mRNA = 11.68, P = 0.0512). Stratification of patients according to tumour staging revealed that *SOCS1* expression was significantly up-regulated in CRC tumour relative to normal tissues in stage II adenocarcinomas (Wilcoxon matched-pairs signed rank test, P = 0.0216), but not in other stages (Fig. 1C). Among the 21 CRC patients with stage II adenocarcinoma, 11 (52%) exhibited above 2-fold increase in *SOCS1* expression in tumours, whereas *SOCS1* under-expression in tumours was denoted in only 2 (9%) patients. Moreover, the median tumour-to-normal ratio of *SOCS1* expression was significantly elevated in stage II and III adenocarcinomas relative to stage I but not in advanced stage IV (Mann Whitney test), (Fig. 1C). Analysis of *SOCS1* relative gene expression in all 431 human CRC patients from the TCGA gene expression datasets showed a significant increase of *SOCS1* mRNA in tumour specimens compared to non-tumour colon tissues [Median *SOCS1* RSEM normalized expression^28^ of 65.29 in normal tissues vs. 107.3 in tumours, Mann Whitney test, P = 0.0105]. Besides, when compared to normal tissues, *SOCS1* expression was found up-regulated in stage I and II colon adenocarcinomas (P = 0.0081 and P = 0.002, respectively, Mann Whitney test), and to a lesser extent in stage III (P = 0.0398), but not in stage IV tumours (Fig. 1D).

We next investigated whether *SOCS1* mRNA expression in CRC tumours correlated with clinicopathological features. For these analyses, CRC patients were stratified based on low and high quartiles of *SOCS1* expression levels in tumours. As shown in Table 1, no significant association was observed between *SOCS1* mRNA expression and patient age, gender, lymphatic and venous invasion, and the degree of differentiation (tumour grade). A weak correlation was seen between low *SOCS1* expression and the presence of lymph node (P = 0.0257, OR = 0.5338) and distant metastasis (P = 0.0109, OR = 0.3326), and early tumour stages (P = 0.0152, OR = 0.5046). However, Kaplan–Meier analysis of the overall survival according to *SOCS1* expression failed to show a significant correlation between patients with high versus low SOCS1 expression (Fig. 1E, Log-rank test, HR = 0.8033, P = 0.5927). Likewise, no relationship between *SOCS1* expression and overall survival was observed when the analysis was carried out with CRC patient subgroups stratified according to stages (data not shown). Overall, these analyses revealed an up-regulation of *SOCS1* expression in tumour relative to normal tissue in the early stage of CRC when compared to more advanced stages of the disease. Notably, they also denoted that *SOCS1* under-expression is relatively rare in CRC, and that altered expression of *SOCS1* is not a reliable predictor of patient CRC survival.

### Establishment of CT26 and CT36 cell models to investigate SOCS1 functions in CRC cells

**Figure 2:**
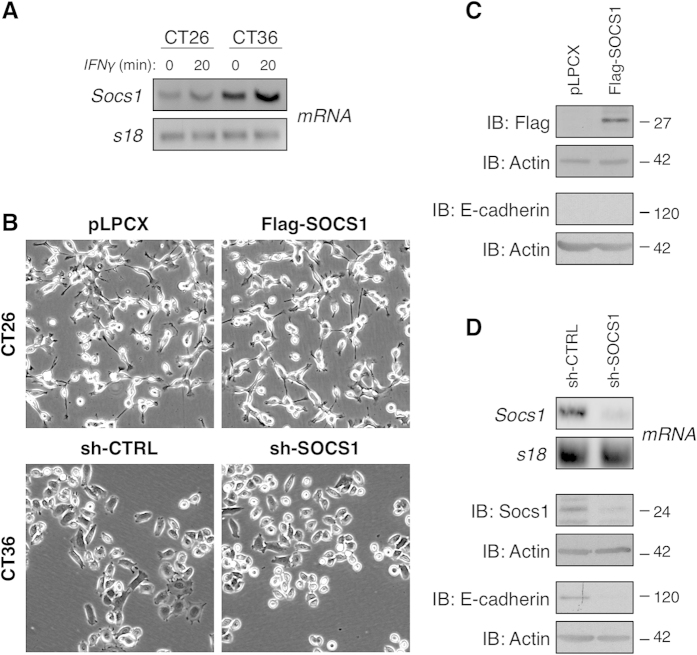
Establishment of CT26 and CT36 CRC cell models to study SOCS1 function in CRC. (**A**) SOCS1 mRNA expression was analysed by semi-quantitative RT-PCR analysis in CT26 and CT36 CRC cells, pre-treated with IFNγ (20 ng/ml) or PBS for 20 min. The mRNA encoding for the S18 ribosomal protein is shown as a control. (**B**) Photographs obtained by phase contrast microscopy (10X magnification) show typical morphology of CT26 cells stably overexpressing Flag-SOCS1 or pLPCX empty vector, and CT36 cells expressing a non-specific random control (sh-CRTL) or SOCS1-targeting shRNAs (sh-SOCS1). (**C**) Ectopic expression of Flag-SOCS1 protein in CT26 cell populations was confirmed by immunoblotting (IB) analyses of total cell lysate (TCL). E-cadherin protein levels were determined. (**D**) Silencing of SOCS1 in CT36 cell populations was validated at the mRNA and the protein levels by RT-PCR and IB analyses, respectively. Concordant with the morphological changes observed upon the knockdown in CT36 cells, E-cadherin protein levels were reduced in CT36 sh-SOCS1 relative to sh-CTRL, as determined by IB analysis. For each IB carried out, actin protein levels provided a loading control. Cropped image of the blots are shown in this figure.

To evaluate the functional relevance of SOCS1 in CRC cells, we have chosen to use the murine CT26 and CT36 colon carcinoma cell lines. These two well-studied and widely used CRC cell lines, derived from carcinogen-induced tumours of BALB/c mice, represent an advantageous system for gain- and loss-of-function studies, since they display markedly opposite cancer features^29^. While the CT26 cells are highly tumourigenic and metastatic, the CT36 cells are devoid of these characteristics. We first evaluated the endogenous expression level of *Socs1* mRNA in these CRC cell lines by semi-quantitative PCR analysis. Because *Socs1* mRNA levels are often minimal at steady-state^30^, cells were treated with interferon (IFN)γ to induce *Socs1* expression. As expected, induction of *Socs1* mRNA upon IFNγ stimulation was observed in both cell lines (Fig. 2A). However, *Socs1* mRNA levels were markedly reduced in the highly tumourigenic and metastatic CT26 cells, relative to the poorly tumourigenic and non-metastatic CT36 cells, with or without IFNγ stimulation (Fig. 2A). These results were concordant with a potential tumour suppressor role for SOCS1 in CRC cells.

In accordance with its expression profile, SOCS1 was overexpressed in the CT26 cells and silenced in the CT36 CRC cells to examine its implication in colorectal carcinogenesis. Populations of CT26 cell stably expressing the *Socs1* gene carrying an upstream 3XFlag epitope (Flag-SOCS1) were generated, along with control CT26 cell populations expressing the pLPCX empty vector. Conversely, CT36 populations stably expressing a shRNA directed against *Socs1* mRNA (sh-SOCS1) or a control non-targeting random sequence (sh-CTRL) were produced. As revealed by phase-contrast microscopy, CT26 cells overexpressing SOCS1 retained their transformed morphological characteristics similar to the control cells, including a spindle shape with limited cell-cell contacts and highly dedifferentiated features (Fig. 2B). By contrast, CT36 cells transduced with a shRNA targeting *Socs1* adopted a slightly less flattened epithelial-like morphology compared to sh-CTRL cells (Fig. 2B). The ectopic expression of Flag-SOCS1 in CT26 cells was confirmed by immunoblotting (IB) with an anti-Flag antibody (Fig. 2C). Knockdown of SOCS1 in CT36 cells was validated by semi-quantitative PCR and IB (Fig. 2D). Concordant with the morphological changes, the protein level of the epithelial marker E-cadherin was reduced in CT36 sh-SOCS1 cells compared to control sh-CTRL cells (Fig. 2D), but unchanged in CT26 CRC cells overexpressing SOCS1 (Fig. 2C).

### SOCS1 promotes pro-tumourigenic functions *in cellulo* and *in vivo* in CT26 and CT36 CRC cells

**Figure 3:**
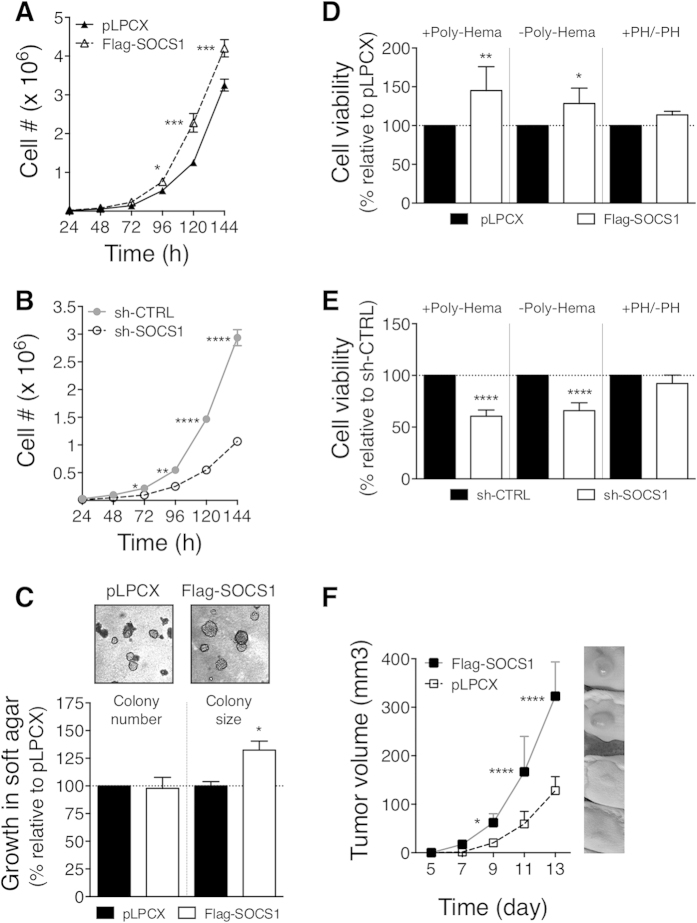
SOCS1 in CT26 and CT36 CRC cells promotes pro-oncogenic responses *in cellulo*, and *in vivo* tumourigenesis in CT26 cells. (**A**,**B**) SOCS1 sustains cellular growth in CT26 and CT36 CRC cells. Cell-count assays were performed after seeding the indicated cell populations under adherent culture conditions in presence of serum. In A, the graph shows representative growth curves of pLPCX and Flag-SOCS1 CT26 cells. In B, growth curves of the sh-CTRL or sh-SOCS1 CT36 cells are shown. (**C**) SOCS1 potentiates anchorage-independent growth in CT26 CRC cells in soft agar. Photographs depict typical morphology of the colonies in soft agar formed by the pLPCX and Flag-SOCS1 CT26 cells. Bar graph shows the average number and size of colonies formed in soft agar. Values are expressed as percentage ± s.e.m. of those produced by CT26 pLPCX cells, calculated from 3 independent experiments performed in triplicate. CT36 sh-CTRL and sh-SOCS1 cells failed to grow in soft agar (data not shown). (**D**,**E**) SOCS1 provided resistance to death signals in CT26 and CT36 CRC cells. The viability of the indicated cell populations was estimated 18 hours after their seeding in suspension or adherent conditions. In D, bar graph shows in percentage the mean ± s.e.m. value of cell viability for the Flag-SOCS1-CT26 relative to that of the CT26 pLPCX cells. In E, bar graph represents the viability of the CT36 sh-SOCS1 cells relative to that of the CT36 sh-CTRL cells. The values were calculated from 3 independent experiments performed in triplicate. (**F**) SOCS1 overexpression in CT26 CRC cells increases tumourigenesis. Growth of tumour (mm^3^) over time was measured after subcutaneous injection of 10^5^ pLPCX or Flag-SOCS1 CT26 cells in BALB/c mice. Results represent the mean ± s.d. tumour volume of n = 5–6.

We next investigated if the modulation of SOCS1 expression had any impact on the cancer features of the CT26 and CT36 cells. According to the reported tumour suppressor role of SOCS1, we were predicting SOCS1 to exert a negative influence on tumour-promoting functions in CRC cells. Surprisingly, the overexpression of SOCS1 in the CT26 cells potentiated cell growth in cell-count assays (Fig. 3A). Inversely, CT36 sh-SOCS1 cells displayed a marked delay in cell growth capacity when compared to the CT36 sh-CTRL cells (Fig. 3B). The capacity of cells to grow in absence of anchorage to the extracellular matrix (ECM) is a typical characteristic associated with cancerous cell malignancy. While the CT26 cells are able to grow in an anchorage-independent manner, as assessed by colony formation in soft agar, the CT36 cells are not^29^. We therefore evaluated whether the modulation of SOCS1 expression in these CRC cells influenced this oncogenic characteristic. Our analyses showed that SOCS1 silencing in the CT36 cells did not alter grow in soft agar relative to the control cells (data not shown). However, while the overexpression of SOCS1 in the CT26 cells did not change the number of colonies formed in soft agar, the size of the colonies produced by CT26 Flag-SOCS1 cells was significantly larger than those produced by control pLPCX cells (Fig. 3C).

We next evaluated the influence of SOCS1 on the capacity of CT26 and CT36 CRC cells to escape cell death induced by ECM detachment (anoikis) and growth factor deprivation. For this, viability of the cells was monitored 18 hours after their seeding in the absence of serum under adherent and suspension conditions [polyhema (PH)-coated plates]. As expected, the viability of CT26 and CT36 cells was markedly diminished in suspension, ranging between 35–50% of survival compared to adherent conditions (data not shown). The CT26 Flag-SOCS1 cells exhibited 48% and 29% increased survival relative to pLPCX control cells when seeded in absence of serum under adherent and non-adherent conditions, respectively (Fig. 3D). Conversely, silencing of SOCS1 in CT36 cells led to a decrease in survival upon serum-starvation when these cells were cultured on plates coated or not with PH (39% and 34%, respectively, Fig. 3E). However, resistance to anoikis conferred by SOCS1 was marginal and non significant in both CT26 and CT36 cells, as determined by the ratio of cell viability in suspension relative to adherent conditions (Fig. 3D,E, +PH/–PH).

These results suggested a pro-oncogenic role of SOCS1 in these CRC cells, rather then its anticipated tumour suppressor function. This was not reflecting the selection of variant clonal cell lines, since our analyses were performed with cell populations. Significantly, SOCS1-mediated effects on cell growth, colony-forming activity in soft agar and resistance to cell death stimuli were mirrored when the expression of SOCS1 was in opposite suppressed in CT26 cells or up-regulated in CT36 cells (Supplementary Fig. 1). Furthermore, SOCS1 overexpression in a rat model of intestinal epithelial cells (IEC-6) transformed by an oncogenic form of the Met receptor (Tpr-Met-IEC6^31^) promoted these same cancer features, in addition to enhance loss of contact inhibition, as determined in focus assays, and migration (Supplementary Fig. 2).

Having shown that SOCS1 mediated pro-oncogenic responses in CT26 CRC cells, we next evaluated whether this translated into enhanced tumourigenic activity *in vivo*. Tumour-forming potential of the CT26 Flag-SOCS1 vs. pLPCX cells was compared following their subcutaneous injection in syngeneic BALB/c mice. The ability of CT36 cells to form tumour *in vivo* was not evaluated since the silencing of SOCS1 in these cells did not confer colony-forming activity in soft-agar. While both CT26 Flag-SOCS1 and control cells formed palpable tumours within a very short latency (~7 days), those derived from Flag-SOCS1-expressing CT26 cells expanded more rapidly than the control cells (Fig. 3F). Taken together, these results provided evidence that SOCS1 can foster tumour-promoting effects in CRC cells.

### SOCS1 limits IFNγ and HGF signalling, enhances p53 activation and down-regulates basal STAT1 protein levels in CRC cells

**Figure 4:**
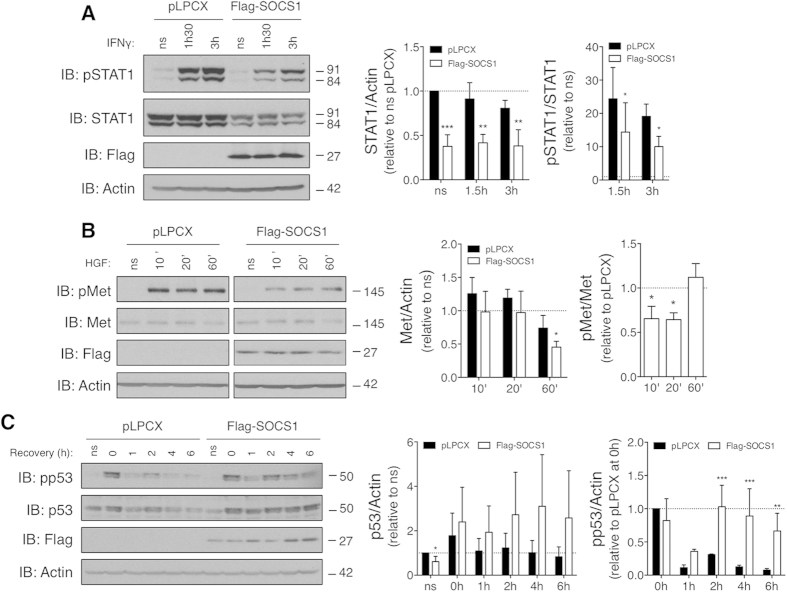
SOCS1 in CT26 CRC cells limits IFNγ- and HGF-induced signalling, prolongs etoposide-driven p53 protein and phosphorylation levels, but reduces steady-state level of STAT1 protein. (**A**) SOCS1 decreases STAT1 expression and limits IFNγ-induced phosphorylation of STAT1. Serum-starved cells were treated with vehicle (PBS) or IFNγ (20 ng/ml) for the indicated time. STAT1 protein and phosphorylation (Tyr701) levels were evaluated by IB analysis of TCL. Quantification was performed by densitometric analysis. The left graph shows fold-change in STAT1 protein levels normalized to actin, relative to non-stimulated (ns) pLPCX control cells. The right graph shows STAT1 phosphorylation levels normalized to total STAT1 levels, relative to ns cells. (**B**) SOCS1 decreases Met expression in CT26 cells in response to HGF-induced sustained activation. Serum-starved cells were stimulated with HGF (25 ng/ml). Met receptor expression and phosphorylation (Tyr1234/1235) levels were determined by IB analysis. The samples were analysed on the same gels and blots. The left graph shows fold-change of Met protein levels normalized to actin relative to ns cells. The right graph shows Met phosphorylation levels normalized to total Met protein relative to pLPCX control cells (Met phosphorylation was undetectable at steady state). (**C**) SOCS1 sustains the expression and activation of p53 induced by etoposide-mediated genotoxic stress. Cells were treated with vehicle (DMSO) or etoposide (100 μM) for two hours, washed with PBS and put back in the presence of medium containing 10% FBS. TCL were prepared after the indicated recovery time points for IB analysis of p53 protein and phosphorylation on Ser18. The left bar graph shows fold-change of p53 protein levels normalized to actin relative to ns cells. The right one shows p53 phosphorylation levels normalized to p53 protein relative to epotoside-treated cells at recovery time 0h (p53 protein phosphorylation was undetected at steady state). All values are expressed as the mean ± s.d. calculated from at least 3 independent experiments. Cropped image of the blots are shown in this figure.

We next sought to determine whether SOCS1 established biological activities were maintained in these CRC cell models. For this, biochemical analyses were performed using the CT26 CRC cells, since the genomic and molecular alterations within these cells has been extensively characterized^32^. To determine whether SOCS1 overexpression inhibited cytokine signalling in CT26 cells, the activation state of STAT1 in response to IFNγ stimulation in Flag-SOCS1 and pLPCX control cells was compared. Strikingly, CT26 cells expressing Flag-SOCS1 exhibited a marked reduction in STAT1 protein levels at both steady state and upon IFNγ stimulation (~50%, Fig. 4A). However, whereas IFNγ stimulation efficiently enhanced STAT1 phosphorylation in both CT26 Flag-SOCS1 and pLPCX cells, but the fold induction of STAT1 phosphorylation induced by IFNγ relative to non-stimulated cells was slightly lower in Flag-SOCS1 expressing CT26 than in the control cells (Fig. 4A). Together, these results indicate that SOCS1 in CT26 CRC cells exerted negative feedback regulation of IFNγ cytokine signalling, but also down-regulated the steady-sate of STAT1 protein level.

In recent studies, we have identified that SOCS1 negatively regulated HGF signalling by promoting ubiquitination and proteasomal degradation of the receptor Met in hepatocytes and HCC cell lines^10^,^11^. To evaluate whether SOCS1 manifested such capacity in CT26 CRC cells, Met expression and activation status in CT26 pLPCX and Flag-SOCS1 cells in response to HGF stimulation were evaluated. As shown by IB analysis, Met protein levels in serum-starved Flag-SOCS1 and pLPCX control CT26 cells did not significantly differ at steady state (Fig. 4B). Concordant with Met being subjected to negative-feedback regulation upon sustained activation, CT26 control cells exhibited reduced Met protein levels in response to 60 min of HGF stimulation. The extent of HGF-mediated Met down-regulation was slightly but significantly potentiated by the expression of Flag-SOCS1 (Fig. 4B). As expected, robust Met tyrosine phosphorylation (Tyr1234/1235) was observed in HGF treated CT26 cells compared to untreated cells. However, Met phosphorylation, relative to its expression levels, diminished in Flag-SOCS1-expressing CT26 cells in response to 10 and 20 min of HGF stimulation, but not after 60 min. Therefore, SOCS1 retained its capacity to inhibit HGF-induced signalling in CT26 cells.

In response to cellular stress such as DNA damage, p53 acts as a tumour suppressor by activating the transcription of genes involved in DNA repair, cell cycle control, senescence and apoptosis^33^. We have previously shown that SOCS1 promoted p53 activity by enhancing its phosphorylation at serine 15 (Ser18 in mice)^12^. As the CT26 cells express wild-type p53^32^,^34^, we therefore examined whether SOCS1 overexpression in these cells potentiated p53 activity in response to the DNA damage-inducing agent, etoposide. For this, CT26 Flag-SOCS1 and pLPCX control cells were treated for two hours with etoposide, and then let to recover in normal growth media for the indicated times prior to assessment of p53 protein and phosphorylation levels. Interestingly, p53 protein levels were slightly reduced at steady state by the expression of Flag-SOCS1 in CT26 cells (Fig. 4C). Nonetheless, etoposide elicited a rapid induction of p53 protein and phosphorylation levels in both CT26 Flag-SOCS1 and control pLPCX cells (Fig. 4C). Notably, more sustained induction of p53 protein and phosphorylation was denoted after etoposide withdrawal in CT26 Flag-SOCS1 cells compared to control pLPCX cells (Fig. 4C). However, the mean fold increase in p53 protein levels over baseline did not reach statistical significance due to the considerable variations between experiments. These results indicated that SOCS1 in CT26 CRC cells was proficient at facilitating p53 activation in response to etoposide-induced genotoxic stress. Collectively, these biochemical analyses showed that although SOCS1 fostered tumour-promoting activity in CT26 CRC cells, it preserved its inhibitory action on cytokine and HGF signalling along with its capacity to enhance p53 activation. Notably, they also identified that SOCS1 decreased the steady-state level of p53 and STAT1 proteins.

### SOCS1 promotes oncogenic responses in human SW620 CRC cells

**Figure 5:**
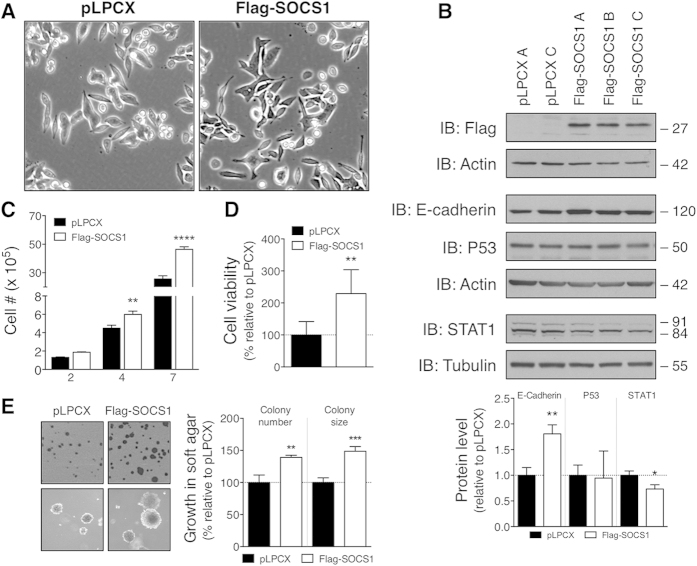
SOCS1 in human SW620 CRC cells promotes pro-oncogenic responses. (**A**) Photographs obtained by phase contrast microscopy (10X magnification) show typical morphology of SW620 cells stably expressing Flag-SOCS1 or pLPCX empty vector. (**B**) Ectopic expression of Flag-SOCS1 protein in independent SW620 cell populations was detected by IB analyses of TCL. E-cadherin, P53 and STAT1 protein levels are also shown, and actin or tubulin protein levels provided loading controls. Cropped image of the blots are shown in this figure. Quantification was performed by densitometric analysis from at least 3 independent experiments. The graph shows the mean ± s.d. fold-change in E-cadherin, P53 or STAT1 protein levels relative to pLPCX control cells normalized to loading control. (**C**) Ectopic expression of Flag-SOCS1 promotes cellular growth in SW620 CRC cells. All functional assays were conducted with pLPCX- and Flag-SOCS1-expressing SW620 cell populations A and C. Cell-counts were performed at the indicated time after seeding the cells in normal adherent culture conditions in triplicate. The histogram shows the mean number of cells  ±  s.e.m., calculated from the independent pLPCX and Flag-SOCS1 SW620 cell populations. (**D**) SOCS1 in SW620 CRC cells promotes resistance to cell death induced by growth factor-deprivation. The viability of cells was estimated by XTT assays 48 hours after their seeding in adherent conditions and absence of serum. Bar graph shows in percentage the mean ± s.e.m. values of cell viability calculated from SW620 cell populations expressing Flag-SOCS1, relative to that of those harbouring pLPCX empty vector. (**E**) SOCS1 potentiates anchorage-independent growth in SW620 CRC cells in soft agar. Photographs depict typical morphology of the colonies in soft agar formed by the pLPCX and Flag-SOCS1 SW620 cells. Bar graph shows the average number and size of colonies formed in soft agar expressed as percentage ± s.e.m., relative to SW620 pLPCX cells from 2 independent cell populations performed in triplicate.

Previous work by others showed that SOCS1 overexpression in human SW620 CRC cells repressed many of their EMT features^22^. However, it was unclear whether or not SOCS1 also evoked pro-oncogenic responses in those cells. To clarify that, we reproduced some of their key experiments but analysed cell populations instead of clonal cell lines. Phase-contrast microscopy showed that Flag-SOCS1-expressing SW620 cell populations were still highly transformed but adopted a slightly more flattened morphology than control cells (Fig. 5A). These morphological changes were associated with a 1.8-fold increase in E-cadherin protein levels in SOCS1-overexpressing SW620 cells (Fig. 5B). Different from the original study^22^, the growth capacity of the SW620 CRC cells was potentiated by the expression of SOCS1 (Fig. 5C), as well as their viability in the absence of serum (Fig. 5D). Furthermore, the expression of SOCS1 enhanced the capacity of these cells to grow in an anchorage-independent manner, as shown by an increase number and size of colonies in soft agar formed by the Flag-SOCS1-expressing SW620 cells when compared to those produced by control pLPCX-transduced cells (Fig. 5E). However, the morphology of the colonies formed by the Flag-SOCS1-expressing SW620 cells and the empty vector was essentially undistinguishable (Fig. 5F). Notably, STAT1 down-regulation was induced by the ectopic expression of SOCS1 in the SW620 cells (Fig. 5B), like observed in rodent CRC cell models (Fig. 4A and Supplementary Figure 1G). In contrast, comparable amounts of P53 protein were detected in Flag-SOCS1 and control SW620 cells (Fig. 5B). Overall, these results confirm the notion that SOCS1 acts as negative regulator of EMT in human SW620 CRC cells, but also support our postulate of a pro-oncogenic role of SOCS1 in CRC.

## Discussion

The relevance of SOCS1 in CRC pathogenesis has been poorly investigated and remains unresolved. In the present study, our analysis of TCGA gene expression datasets of matched tumour/normal human CRC specimens reveals that *SOCS1* expression is more frequently up-regulated than reduced in CRC primary tumours (Fig. 1A). Overexpression of *SOCS1* in tumour relative to the paired normal mucosa is predominant in stage II adenocarcinomas (Fig. 1C). Likewise, *SOCS1* highest relative expression in tumours is prevalent in early-stage I and II adenocarcinomas (Fig. 1D). These results are somewhat consistent with the analysis of David *et al.*^22^, in which a small cohort of human CRC samples revealed the highest *SOCS1* mRNA and protein levels in normal colon and stage I and II adenomas. In contrast, while they showed reduced expression of SOCS1 protein in poorly differentiated carcinomas, we found no significant association between *SOCS1* mRNA expression and tumour grade in the TCGA cohort (Table 1). Nonetheless, low *SOCS1* expression in cancer specimens showed a weak but significant correlation with lymph node and distant metastasis, and tumour stages of the disease (Table 1). However, in line with Ayyildiz *et al.* previous report^23^, our analyses failed to reveal a positive correlation between high *SOCS1* mRNA levels in CRC tumours and better overall patient survival, even upon CRC patient stratification according to stages (Fig. 1E). Taken altogether, these clinical observations show that *SOCS1* expression has no prognostic significance in CRC.

However, our study identify, for the first time, that SOCS1 possesses tumour-promoting activity in CRC cells. Namely, SOCS1 overexpression in CT26 or CT36 CRC cells enhanced cell growth, anchorage-independent growth and resistance to cell death stimuli, while its silencing inhibited these tumor-promoting features (Figs 2 and 3; and Supplementary Fig. 1). Furthermore, SOCS1 overexpression boosted the tumourigenic potential of the mouse CT26 CRC cells *in vivo* (Fig. 3). These oncogenic responses were potentiated by the ectopic expression of SOCS1 in Met-transformed rat intestinal epithelial cell model (Supplementary Fig. 2) and human metastatic SW620 CRC cells (Fig. 5). Our findings are in conflict with the only published report that has investigated SOCS1-regulated functions in CRC cells, in which the overexpression of SOCS1 did not impact the proliferation and anchorage-independent growth of SW620 CRC cells^22^. In that work, the biological influence of SOCS1 expression in SW620 cells was evaluated following the selection of individual clonal cell lines, where instead we have used uncloned cell populations. It is plausible that SOCS1 pro-oncogenic activity was concealed by the selection of clonal cell lines with distinct characteristics to the parental uncloned SW620 cell population. Nonetheless, as the original study, we show that overexpression of SOCS1 in SW620 cells up-regulated the expression of the EMT marker E-cadherin along with inducing a modest morphologic change from fibroblastic to epithelial shape (Fig. 5). These EMT-linked features were reduced when the expression of SOCS1 was silenced in the poorly transformed CT36 (Fig. 2). In the CT26 cells, ectopic expression of Flag-SOCS1 did not induced any reversion in their transformed morphology (Fig. 2), which maybe reflecting the highly degree of transformation of these cells. Taken all together, our results substantiate a role for SOCS1 as a negative regulator of EMT pathways^22^, and provide compelling evidence of a tumour-promoting role of SOCS1 in CRC cells. In the light of the apparent distinctive abundance of SOCS1 in CRC according to stages, that is, elevated expression of SOCS1 in primary tumours at early stages but reduced expression in advanced stages, this raises the interesting possibility that SOCS1 may have stage-specific functions and therefore, implications for the clinical course of the disease.

Our findings that SOCS1 promoted pro-oncogenic functions in CRC cells sharply contrast with those reported in our earlier investigations in hepatocytes and HCC cell lines, where SOCS1 suppressed tumourigenic and metastatic biological responses^5^,^10^,^11^. However, a number of studies indicate that SOCS1 can operate as a tumour-promoting protein. For instance, SOCS1 was shown to confer resistance to anti-cancer actions of IFNs in a cell-autonomous manner by limiting their anti-proliferative and pro-apoptotic effects in cancer cells^35^,^36^,^37^,^38^. In murine melanoma cells, which constitutively express SOCS1, its silencing by shRNA was shown to significantly reduce the tumourigenic and metastatic features of these cells *in cellulo* and *in vivo*^16^. Other illustrations of SOCS1 pro-oncogenic activity include its critical role in promoting FGFR-induced activation of the mitogenic MAPK pathway in human chondrocytes, and hedgehog-driven anchorage-independent growth in human keratinocytes and medulloblastomas^14^,^39^. Despite previous observations of SOCS1 pro-oncogenic functions in various cancer cell types, the molecular mechanisms remained undefined.

Structure-function analyses have established that negative feedback regulation of cytokine-induced activation of STATs by SOCS1 involves coordinated binding of its central SH2 domain to JAK tyrosine kinases and inhibition of their catalytic activity by the SOCS1 kinase-inhibitory region (KIR)^4^,^5^. On the other hand, the activation of p53 by SOCS1 requires a direct interaction between the SH2 domain of SOCS1 and p53, and the binding of its C-terminal SOCS Box domain to the ATM/ATR kinases^12^. An interaction between the SOCS1 SH2 and the activated Met receptor, along with the integrity of SOCS1 E3 ubiquitin ligase activity are required for SOCS1-mediated ubiquitination and proteasomal degradation of activated Met receptor^11^. Our biochemical analyses show that SOCS1 can still enhance p53 protein expression and phosphorylation in response to genotoxic stress in CT26 cells (Fig. 4). Likewise, SOCS1 maintains its capacity to limit HGF-mediated Met receptor expression and IFNγ-induced STAT1 phosphorylation in these cells (Fig. 4). These results suggest that SOCS1 tumour-promoting effect in these CRC cells is unlikely to stem from an overall loss in the integrity of its SH2 or SOCS box domains, or invalidation of its E3 ubiquitin ligase activity. In addition to these critical domains, SOCS1 contains two proline-rich motifs in its N-terminal region mediating interactions with SH3-containing proteins, including Grb2 and p85 subunit of the PI3K^4^,^5^. Detailed structure-function analyses will be necessary to define the structural determinants of SOCS1 tumour-promoting activity in CRC cells, as well as additional studies to elucidate the precise molecular mechanisms. Nonetheless, our biochemical analyses suggest that SOCS1 oncogenic activity involves p53-independent pathways, since down-regulation of P53 protein level was promoted by SOCS1 in the CT26 CRC cells but not in the SW620 CRC cells (Figs 4 and 5). In preliminary analyses, basal phosphorylation and expression of ERK and AKT were unaltered by the overexpression or silencing of SOCS1 in CT26 and CT36 CRC cells (data not shown), suggesting that activation of these oncogenic pathways is not underlying the pro-oncogenic effect of SOCS1 in CRC cells. However, the pro-oncogenic activity of SOCS1 in the murine CT26 and CT36 cells, as well in the human SW620 CRC cells coincided with a marked down-regulation of STAT1 protein levels at steady state (Figs 4 and 5, and Supplementary Fig. 1). The activation of STAT1-dependent pathways is known for its promoting anti-proliferative and pro-apoptotic cellular effects^40^,^41^. Furthermore, high STAT1 activity in human colorectal carcinoma specimens was recently identified as a favourable clinical prognosis^42^. These premises suggest that SOCS1-mediated inhibition of STAT1 could therefore contribute to its pro-oncogenic activity in our CRC cell models, which warrant further investigation.

Seminal studies have demonstrated that a same cancer-related protein, acting as either dominant pro- or anti-oncoprotein, can enact cell-type-specific dichotomous functions, or even within the same cell type^43^,^44^,^45^,^46^. The genetic cellular context has emerged as a determinant factor in the paradoxical activities of these cancer proteins^43^,^44^. As CRC progression concurs with the accumulation of multiple heterogeneous genetic alterations^47^, it is possible that specific intrinsic genetic and molecular alterations within CRC cancer cells dictate whether SOCS1 operates as either an oncogene or tumour suppressor in CRC. Our analysis of TCGA gene expression datasets for a large cohort of human CRC specimens, where *SOCS1* expression level did not allow prognostic stratification of CRC patients provides clinical reinforcement to this idea. Comprehensive understanding of the mechanisms underlying SOCS1 tumour-promoting vs. tumour suppressor activity in cancer cells may unravel the molecular “switch” dictating whether SOCS1 promotes pro-oncogenic or tumour suppressor functions. These novel insights will foster a paradigm shift in our understanding of SOCS1 regulated functions not only limited to CRC, but to other types of cancer as well.

## Methods

### Antibodies, reagents and immunoblotting

The polyclonal SOCS1 antibody that was raised in rabbit against the carboxyl-terminus peptide of human SOCS1 was purchased from Abcam (Toronto, ON, Canada). The β-Actin and Flag monoclonal antibodies were purchased from Sigma-Aldrich Canada (Oakville, ON, Canada). The p53, pp53 (Ser15, equivalent to Ser18 in mouse), pSTAT1 (Tyr701) and pMet (Tyr1234/1235) antibodies were acquired from Cell Signaling Technology (Danvers, MA, USA). The STAT1 antibody was obtained from Santa Cruz Technology (Santa Cruz, CA, USA). The E-cadherin antibody was purchased from BD Transduction Labs (Lexington, KY, USA). The Met 148 antibody was kindly provided by Morag Park (McGill University, Montreal, QC, Canada). Anti-mouse IgG HRP-linked and Protein A HRP-linked were used as secondary antibodies and were purchased from GE Healthcare (Piscataway NJ, USA). Murine HGF and IFNγ were purchased from PeproTech (Rocky Hill, NJ, USA) and etoposide was obtained from Sigma Aldrich Canada. Preparation of TCLs, SDS-PAGE and IB analysis were performed as previously described^31^,^48^.

### Cell culture

The CT26 and CT36 cell lines were obtained from Nicole Beauchemin (McGill University, QC, Canada). Authentication of the CT26 and CT36 cells, on the basis of their distinctive tumourigenic and metastatic characteristics *in cellulo* (morphology, E-cadherin expression, colony-forming activity in soft-agar) or *in vivo* (tumour and metastases formation in syngeneic Balb/c mice)^29^, is of routine in our laboratory. The human SW620 colorectal adenocarcinoma cells were from ATCC. These cells were certified negative for mycoplasma. Cell populations stably expressing pLPCX or Flag-SOCS1 cDNAs^49^ were generated by retroviral infections as described previously^31^. Those harbouring pLKO-sh-CTRL or sh-SOCS1 expression cassettes (Sigma Aldrich, Oakville, ON, Canada) were produced by lentiviral infections^48^. All cell populations were expanded from a pool of at least 50 puromycin-resistant colonies (2 μg/ml, Wisent, St-Bruno, QC, Canada), and were then maintained in DMEM containing 10% foetal bovine serum (FBS), 50 μg/ml gentamicin (Wisent) and 2 μg/ml puromycin. For all experiments, each cell population and its associated control had comparable number of passages (±2) and a range between 11 to 25 passages. For cytokine signalling analysis, cells were stimulated with IFNγ at 20 ng/ml or PBS following serum starvation overnight. For Met receptor activation, cells were serum starved overnight and stimulated with HGF used at a concentration of 25 ng/ml. To examine p53 activity in response to genotoxic stress, the cells were pulsed by a treatment with etoposide at 100 μM or DMSO for 2 hours, and then allowed to recover in normal growth media for the indicated time. Unless otherwise indicated, biochemical analyses were performed at least three times with independent lysate preparation of cells.

### Cell-count, soft agar growth, survival and anoikis assays

Cell-count and soft agar assays were performed as previously described^31^,^48^. Briefly, cells were seeded at a density of 2.5–5 × 10^4^ for the cell growth assays and daily cell counts were performed. For soft agar assays, 5 000 cells were embedded in Noble Agar (Difco) and colonies were counted 7–28 days after seeding. For survival and anoikis assays, cells were seeded in Opti-MEM without serum (Invitrogen) at a density of 1.25 × 10^5^ cells/well in 24-well plates that were pre-coated or not with polyHEMA [poly-(2-hydroxyethyl methacrylate); Sigma]. After 18 hours, viability of cells seeded in polyHEMA-coated wells and in non-coated wells was determined by colorimetric XTT methods. Unless otherwise indicated, biological assays were performed in triplicate and at least three times.

### In vivo tumourigenesis

Tumourigenesis assays were performed essentially as previously described^31^,^50^ with 4- to 5-wk-old female BALB/c mice (Charles River, Burlington, MA, USA) under protocols approved by the Université de Sherbrooke Ethics Committee for Animal Care and Use in accordance with guidelines established by the Canadian Council on Animal Care (Protocol ID number 255-14). Briefly, 10^5^ cells/100 μl of DMEM were injected subcutaneously into mice. Tumour volumes were then measured periodically.

### TCGA gene expression data

The expression of *SOCS1* in human CRC specimens was analysed using publicly available TCGA Illumina RNA-Seq datasets of 431 CRC patients linked with their clinical parameters and follow-up data information^27^ (http://cancergenome.nih.gov). Amongst these, matched tumour and non-tumour specimens were available for 41 CRC patients.

### Statistics

Statistical significance difference between means or medians was evaluated by two-tailed t-test, one sample t-test or Wilcoxon rank test. Chi-squared test was used for the association between two categorical groups. Overall survival analyses were determined by Kaplan-Meier method, where the difference was evaluated by the Log-rank test. Statistical analysis was performed with GraphPad Prism v.6 Software (San Diego, CA, USA). The symbol *indicates statistical significance with a P-value ≤ 0.05; **P ≤ 0.01; ***P ≤ 0.001 and ****P ≤ 0.0001.

## Additional Information

**How to cite this article**: Tobelaim, W. S. *et al.* Tumour-promoting role of SOCS1 in colorectal cancer cells. *Sci. Rep.* **5**, 14301; doi: 10.1038/srep14301 (2015).

## Supplementary Material

Supplemental Figures

## Figures and Tables

**Table 1:** Association of *SOCS1* mRNA expression with clinicopathological parameters.

Features	Number of patients	*SOCS1* expression		P value
		Low	High	
Age (years)				
<65	88	51 (58.0%)	37 (42.0%)	0.0518
>65	126	56 (44.4%)	70 (55.6%)	
Gender				
Male	117	58 (49.6%)	59 (50.4%)	0.7834
Female	96	49 (51.0%)	47 (49.0%)	
Lymphatic invasion				
No	111	54 (48.6%)	57 (51.4%)	0.5331
Yes	75	33 (44.0%)	42 (56.0%)	
Venous invasion				
No	136	68 (50.0%)	68 (50.0%)	0.404
Yes	40	17 (42.5%)	23 (57.5%)	
Tumour grade				
T1, T2	40	17 (42.5%)	23 (57.5%)	0.2928
T3, T4	174	90 (51.7%)	84 (48.3%)	
Lymph node metastasis				
N0	128	56 (43.8%)	72 (56.3%)	0.0257*
N1, N2	86	51 (59.3%)	35 (40.7%)	
Distant metastasis				
M0	163	74 (45.4%)	89 (54.6%)	0.0109*
M1	28	20 (71.4%)	8 (28.6%)	
Tumour stage				
I, II	122	52 (42.6%)	70 (57.4%)	0.0152*
III, IV	89	53 (59.6%)	36 (40.4%)	

Data are given as number and percentage (in brackets) based on the total number of patients with *SOCS1* high and low expressing tumours. P values were determined by Chi-square test.

## References

[b1] Elliott J., Hookham M. B. & Johnston J. A. The suppressors of cytokine signalling E3 ligases behave as tumour suppressors. Biochem Soc Trans 36, 464–8 (2008). 18481982 10.1042/BST0360464

[b2] Zhang J., Li H., Yu J. P., Wang S. E. & Ren X. B. Role of SOCS1 in tumor progression and therapeutic application. Int J Cancer 130, 1971–80 (2012). 22025331 10.1002/ijc.27318

[b3] Inagaki-Ohara K., Kondo T., Ito M. & Yoshimura A. SOCS, inflammation, and cancer. Jak-Stat 2, e24053 (2013). 24069550 10.4161/jkst.24053 PMC3772102

[b4] Linossi E. M., Babon J. J., Hilton D. J. & Nicholson S. E. Suppression of cytokine signaling: The SOCS perspective. Cytokine Growth Factor Rev 24, 241–8 (2013). 23545160 10.1016/j.cytogfr.2013.03.005 PMC3816980

[b5] Gui Y. *et al.* Regulation of MET receptor signaling by SOCS1 and its implications for hepatocellular carcinoma. Curr Pharm Des 20, 2922–33 (2014). 23944359 10.2174/13816128113199990597

[b6] Kazi J. U., Kabir N. N., Flores-Morales A. & Ronnstrand L. SOCS proteins in regulation of receptor tyrosine kinase signaling. Cell Mol Life Sci 71, 3297–310 (2014). 24705897 10.1007/s00018-014-1619-y PMC11113172

[b7] Yoshikawa H. *et al.* SOCS-1, a negative regulator of the JAK/STAT pathway, is silenced by methylation in human hepatocellular carcinoma and shows growth-suppression activity. Nat Genet 28, 29–35 (2001). 11326271 10.1038/ng0501-29

[b8] Um T. H. *et al.* Aberrant CpG island hypermethylation in dysplastic nodules and early HCC of hepatitis B virus-related human multistep hepatocarcinogenesis. J Hepatol 54, 939–47 (2011). 21145824 10.1016/j.jhep.2010.08.021

[b9] Nishida N., Kudo M., Nagasaka T., Ikai I. & Goel A. Characteristic patterns of altered DNA methylation predict emergence of human hepatocellular carcinoma. Hepatology 56, 994–1003 (2012). 22407776 10.1002/hep.25706

[b10] Gui Y. *et al.* SOCS1 controls liver regeneration by regulating HGF signaling in hepatocytes. J Hepatol 55, 1300–8 (2011). 21703184 10.1016/j.jhep.2011.03.027

[b11] Gui Y. *et al.* Regulation of MET receptor tyrosine kinase signaling by suppressor of cytokine signaling 1 in hepatocellular carcinoma. Oncogene, 10.1038/onc.2015.20 (2015). 25728680

[b12] Calabrese V. *et al.* SOCS1 links cytokine signaling to p53 and senescence. Mol Cell 36, 754–67 (2009). 20005840 10.1016/j.molcel.2009.09.044

[b13] Raccurt M. *et al.* Suppressor of cytokine signalling gene expression is elevated in breast carcinoma. Br J Cancer 89, 524–32 (2003). 12888825 10.1038/sj.bjc.6601115 PMC2394374

[b14] Laner-Plamberger S. *et al.* Hedgehog/GLI signaling activates suppressor of cytokine signaling 1 (SOCS1) in epidermal and neural tumor cells. PLoS One 8, e75317 (2013). 24058673 10.1371/journal.pone.0075317 PMC3769249

[b15] Li Z. *et al.* Expression of SOCS-1, suppressor of cytokine signalling-1, in human melanoma. J Invest Dermatol 123, 737–45 (2004). 15373779 10.1111/j.0022-202X.2004.23408.x

[b16] Scutti J. A. *et al.* Role of SOCS-1 Gene on Melanoma Cell Growth and Tumor Development. Transl Oncol 4, 101–9 (2011). 21461173 10.1593/tlo.10250 PMC3069653

[b17] Nosho K. *et al.* Comprehensive biostatistical analysis of CpG island methylator phenotype in colorectal cancer using a large population-based sample. PLoS One 3, e3698 (2008). 19002263 10.1371/journal.pone.0003698 PMC2579485

[b18] Lee S., Cho N. Y., Yoo E. J., Kim J. H. & Kang G. H. CpG island methylator phenotype in colorectal cancers: comparison of the new and classic CpG island methylator phenotype marker panels. Arch Pathol Lab Med 132, 1657–65 (2008). 18834226 10.5858/2008-132-1657-CIMPIC

[b19] Ogino S. *et al.* Evaluation of markers for CpG island methylator phenotype (CIMP) in colorectal cancer by a large population-based sample. J Mol Diagn 9, 305–14 (2007). 17591929 10.2353/jmoldx.2007.060170 PMC1899428

[b20] Ogino S., Kawasaki T., Kirkner G. J., Loda M. & Fuchs C. S. CpG island methylator phenotype-low (CIMP-low) in colorectal cancer: possible associations with male sex and KRAS mutations. J Mol Diagn 8, 582–8 (2006). 17065427 10.2353/jmoldx.2006.060082 PMC1876166

[b21] Samowitz W. S. *et al.* Evaluation of a large, population-based sample supports a CpG island methylator phenotype in colon cancer. Gastroenterology 129, 837–45 (2005). 16143123 10.1053/j.gastro.2005.06.020

[b22] David M. *et al.* Suppressor of cytokine signaling 1 modulates invasion and metastatic potential of colorectal cancer cells. Molecular oncology 8, 942–55 (2014). 24726456 10.1016/j.molonc.2014.03.014 PMC5528518

[b23] Ayyildiz T., Dolar E., Adim S. B., Eminler A. T. & Yerci O. Lack of Prognostic Significance of SOCS-1 Expression in Colorectal Adenocarcinomas. Asian Pac J Cancer Prev 15, 8469–74 (2014). 25339048 10.7314/apjcp.2014.15.19.8469

[b24] Hanada T. *et al.* IFNgamma-dependent, spontaneous development of colorectal carcinomas in SOCS1-deficient mice. J Exp Med 203, 1391–7 (2006). 16717119 10.1084/jem.20060436 PMC2118311

[b25] Hashimoto M. *et al.* Silencing of SOCS1 in macrophages suppresses tumor development by enhancing antitumor inflammation. Cancer Sci 100, 730–6 (2009). 19469017 10.1111/j.1349-7006.2009.01098.x PMC11159406

[b26] Shen L., Evel-Kabler K., Strube R. & Chen S. Y. Silencing of SOCS1 enhances antigen presentation by dendritic cells and antigen-specific anti-tumor immunity. Nat Biotechnol 22, 1546–53 (2004). 15558048 10.1038/nbt1035

[b27] Cancer Genome Atlas N. Comprehensive molecular characterization of human colon and rectal cancer. Nature 487, 330–7 (2012). 22810696 10.1038/nature11252 PMC3401966

[b28] Li B. & Dewey C. N. RSEM: accurate transcript quantification from RNA-Seq data with or without a reference genome. BMC bioinformatics 12, 323 (2011). 21816040 10.1186/1471-2105-12-323 PMC3163565

[b29] Brattain M. G., Strobel-Stevens J., Fine D., Webb M. & Sarrif A. M. Establishment of mouse colonic carcinoma cell lines with different metastatic properties. Cancer Res 40, 2142–6 (1980). 6992981

[b30] Krebs D. L. & Hilton D. J. SOCS proteins: negative regulators of cytokine signaling. Stem Cells 19, 378–87 (2001). 11553846 10.1634/stemcells.19-5-378

[b31] Bernier J., Chababi W., Pomerleau V. & Saucier C. Oncogenic engagement of the Met receptor is sufficient to evoke angiogenic, tumorigenic, and metastatic activities in rat intestinal epithelial cells. Am J Physiol Gastrointest Liver Physiol 299, G677–86 (2010). 20539003 10.1152/ajpgi.00315.2009

[b32] Castle J. C. *et al.* Immunomic, genomic and transcriptomic characterization of CT26 colorectal carcinoma. BMC Genomics 15, 190 (2014). 24621249 10.1186/1471-2164-15-190 PMC4007559

[b33] Lowe S. W., Cepero E. & Evan G. Intrinsic tumour suppression. Nature 432, 307–15 (2004). 15549092 10.1038/nature03098

[b34] Luo J. L., Maeda S., Hsu L. C., Yagita H. & Karin M. Inhibition of NF-kappaB in cancer cells converts inflammation- induced tumor growth mediated by TNFalpha to TRAIL-mediated tumor regression. Cancer Cell 6, 297–305 (2004). 15380520 10.1016/j.ccr.2004.08.012

[b35] Fojtova M. *et al.* Development of IFN-gamma resistance is associated with attenuation of SOCS genes induction and constitutive expression of SOCS 3 in melanoma cells. Br J Cancer 97, 231–7 (2007). 17579625 10.1038/sj.bjc.6603849 PMC2360293

[b36] Takahashi Y. *et al.* Enhancement of antiproliferative activity of interferons by RNA interference-mediated silencing of SOCS gene expression in tumor cells. Cancer Sci 99, 1650–5 (2008). 18754879 10.1111/j.1349-7006.2008.00850.x PMC11158848

[b37] Zitzmann K. *et al.* SOCS1 silencing enhances antitumor activity of type I IFNs by regulating apoptosis in neuroendocrine tumor cells. Cancer Res 67, 5025–32 (2007). 17510435 10.1158/0008-5472.CAN-06-2575

[b38] Guenterberg K. D. *et al.* Enhanced anti-tumor activity of interferon-alpha in SOCS1-deficient mice is mediated by CD4(+) and CD8(+) T cells. Cancer Immunol Immunother 60, 1281–8 (2011). 21604070 10.1007/s00262-011-1034-2 PMC3521522

[b39] Ben-Zvi T., Yayon A., Gertler A. & Monsonego-Ornan E. Suppressors of cytokine signaling (SOCS) 1 and SOCS3 interact with and modulate fibroblast growth factor receptor signaling. Journal of cell science 119, 380–7 (2006). 16410555 10.1242/jcs.02740

[b40] Avalle L., Pensa S., Regis G., Novelli F. & Poli V. STAT1 and STAT3 in tumorigenesis: A matter of balance. Jak-Stat 1, 65–72 (2012). 24058752 10.4161/jkst.20045 PMC3670295

[b41] Klampfer L. The role of signal transducers and activators of transcription in colon cancer. Front Biosci 13, 2888–99 (2008). 17981761 10.2741/2893

[b42] Gordziel C., Bratsch J., Moriggl R., Knosel T. & Friedrich K. Both STAT1 and STAT3 are favourable prognostic determinants in colorectal carcinoma. Br J Cancer 109, 138–46 (2013). 23756862 10.1038/bjc.2013.274 PMC3708576

[b43] Stepanenko A. A., Vassetzky Y. S. & Kavsan V. M. Antagonistic functional duality of cancer genes. Gene 529, 199–207 (2013). 23933273 10.1016/j.gene.2013.07.047

[b44] Durbin A. D., Hannigan G. E. & Malkin D. Oncogenic ILK, tumor suppression and all that JNK. Cell Cycle 8, 4060–6 (2009). 19923885 10.4161/cc.8.24.10093

[b45] Musteanu M. *et al.* Stat3 is a negative regulator of intestinal tumor progression in Apc(Min) mice. Gastroenterology 138, 1003-11 e1–5 (2010). 19962983 10.1053/j.gastro.2009.11.049

[b46] Nguyen A. V. *et al.* STAT3 in epithelial cells regulates inflammation and tumor progression to malignant state in colon. Neoplasia 15, 998–1008 (2013). 24027425 10.1593/neo.13952 PMC3769879

[b47] Arends J. W. Molecular interactions in the Vogelstein model of colorectal carcinoma. J Pathol 190, 412–6 (2000). 10699988 10.1002/(SICI)1096-9896(200003)190:4<412::AID-PATH533>3.0.CO;2-P

[b48] Pomerleau V., Landry M., Bernier J., Vachon P. H. & Saucier C. Met receptor-induced Grb2 or Shc signals both promote transformation of intestinal epithelial cells, albeit they are required for distinct oncogenic functions. BMC Cancer 14, 240 (2014). 24708867 10.1186/1471-2407-14-240 PMC4234027

[b49] Mallette F. A., Calabrese V., Ilangumaran S. & Ferbeyre G. SOCS1, a novel interaction partner of p53 controlling oncogene-induced senescence. Aging (Albany NY) 2, 445–52 (2010). 20622265 10.18632/aging.100163 PMC2933891

[b50] Saucier C. *et al.* The Shc adaptor protein is critical for VEGF induction by Met/HGF and ErbB2 receptors and for early onset of tumor angiogenesis. Proc Natl Acad Sci USA 101, 2345–50 (2004). 14983012 10.1073/pnas.0308065101 PMC356953

