# Supplementary material for: Tumour-promoting role of SOCS1 in colorectal cancer cells
Source: Sci Rep. 2015 Sep 22;5:14301. doi: 10.1038/srep14301 (PMC4585755; doi:10.1038/srep14301)
Supplement: Supplemental Figures [file srep14301-s1.pdf]

Supplementary information

Tumour-promoting role of SOCS1 in colorectal cancer cells

William S. Tobelaim[1,†], Claudia Beaurivage[1,†], Audrey Champagne[1], Véronique Pomerleau[1], Aline Simoneau[1], Walid Chababi[1], Mehdi Yeganeh[2], Philippe Thibault[3], Roscoe Klinck[3], Julie C. Carrier[1], Gerardo Ferbeyre[4], Subburaj Ilangumaran[2,¶] and Caroline Saucier[1,¶,*]

* To whom correspondence should be addressed. Tel: 1 819 821 8000 x75279; Fax: 1 819 820 6831; Email: caroline.saucier@usherbrooke.ca

† These authors contributed equally to this work.

¶ These authors jointly supervised this work.

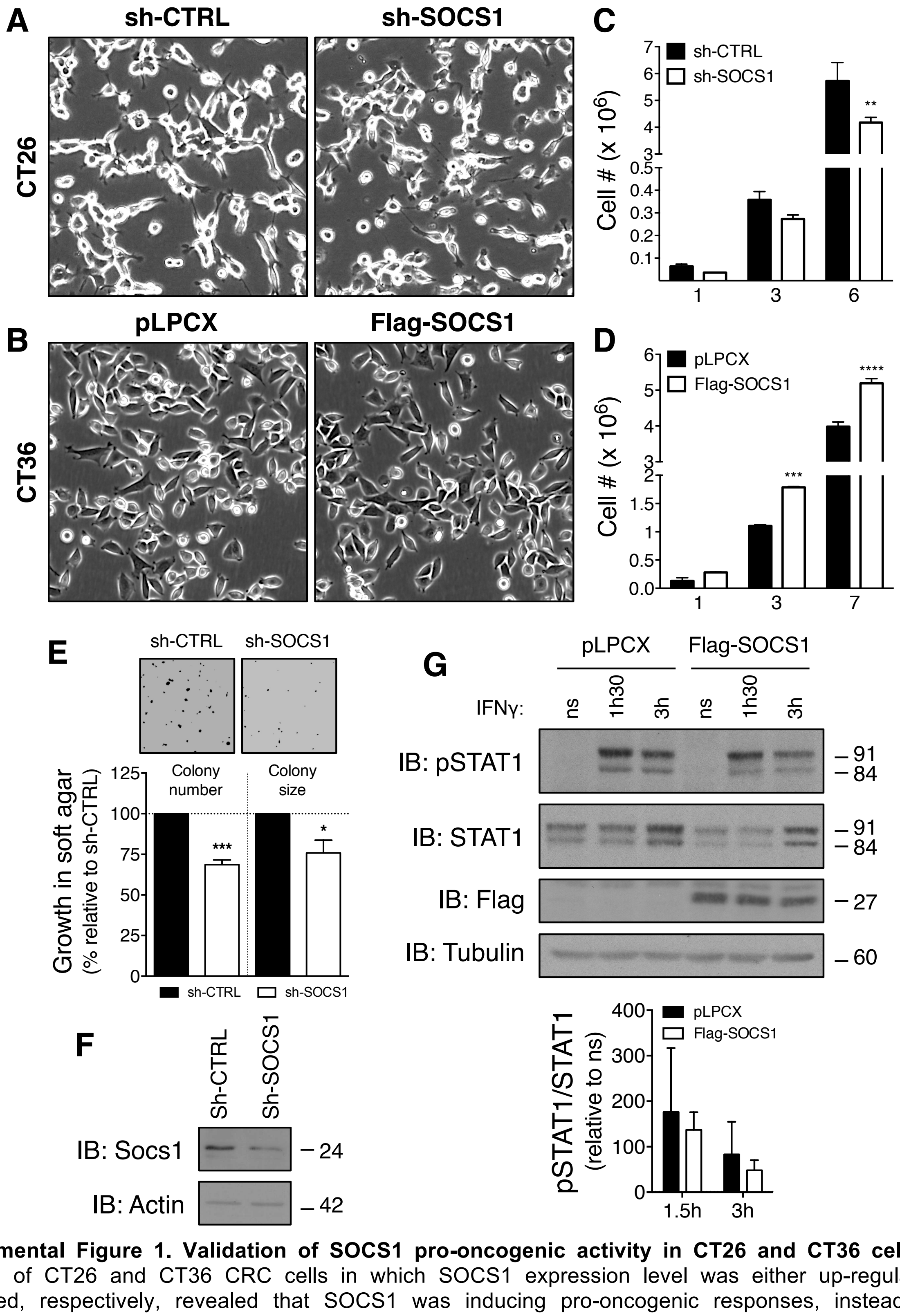

Supplemental Figure 1. Validation of SOCS1 pro-oncogenic activity in CT26 and CT36 cells. Our analysis of CT26 and CT36 CRC cells in which SOCS1 expression level was either up-regulated or repressed, respectively, revealed that SOCS1 was inducing pro-oncogenic responses, instead of its anticipated tumour suppressing effect. We therefore set out to duplicate key experiments in CT26 cells where SOCS1 was silenced, and in CT36 cells in which SOCS1 was overexpressed. Populations of CT36 cells stably expressing pLPCX or Flag-SOCS1 cDNAs were generated by retroviral infections. Those of CT26 cells harbouring pLKO-sh-CTRL or sh-SOCS1 expressing cassette were produced by lentiviral infections. (**A**) Photographs obtained by phase contrast microscopy (10X magnification) show typical morphology of sh-CTRL and sh-SOCS1 CT26 cells. Compared to control CT26 cells harbouring a non-targeting scrambled RNA sequence, those transduced with an shRNA targeting *Socs1* displayed similar transformed morphological characteristics. (**B**) Photographs show the morphology of the CT36 Flag-SOCS1 or pLPCX cells. Overexpression of SOCS1 did not overtly change the morphology of the CT36 cells, except inducing a very subtle more flatted appearance compared to the control cells. (**C** and **D**) SOCS1 sustains

cellular growth in CT26 and CT36 CRC cells. Cell-counts were performed at the indicated time after seeding the cells under adherent culture conditions in presence of serum. The histogram shows the mean number of cells ± s.e.m. calculated from 3 independent experiments done in triplicate. While the silencing of SOCS1 in CT26 cells reduced their growth capacity, its overexpression in CT36 cells boosted cell growth. (**E**) Silencing of SOCS1 reduces anchorage-independent growth in CT26 CRC cells. Photographs show the colonies in soft agar formed by the sh-CTRL and sh-SOCS1 CT26 cells. Bar graph shows the average number and size of colonies formed in soft agar. Values are expressed as percentage ± s.e.m. of those produced by sh-CTRL CT26 cells, calculated from 3 independent experiments performed in triplicate. (**F**) Validation of SOCS1 silencing in sh-SOCS1 CT26 cell populations by IB analyses. (**G**) SOCS1 overexpression in CT36 cells down-regulates STAT1 protein levels. Serum-starved cells were treated with vehicle (PBS) or stimulated IFNγ with (20 ng/ml) for the indicated time. STAT1 protein and phosphorylation (Tyr701) levels, and Flag-SOCS1 expression were evaluated by IB of TCL. Quantification was performed by densitometric analysis. The graph shows STAT1 phosphorylation levels normalized to total STAT1 levels, relative to non-stimulated cells. The values are expressed as the mean ± s.d. calculated from at least 2 independent experiments. A 62.9% ± 10.2 (P = 0.0012, n=4) reduction in STAT1 protein levels (normalized to actin or tubulin) was revealed at steady state in Flag-SOCS1 CT36 cells relative to control pLPCX cells. Overall, these results provide further validation for the pro-oncogenic activity SOCS1 in CT26 and CT36 CRC cells. Cropped image of the blots are shown in this figure.

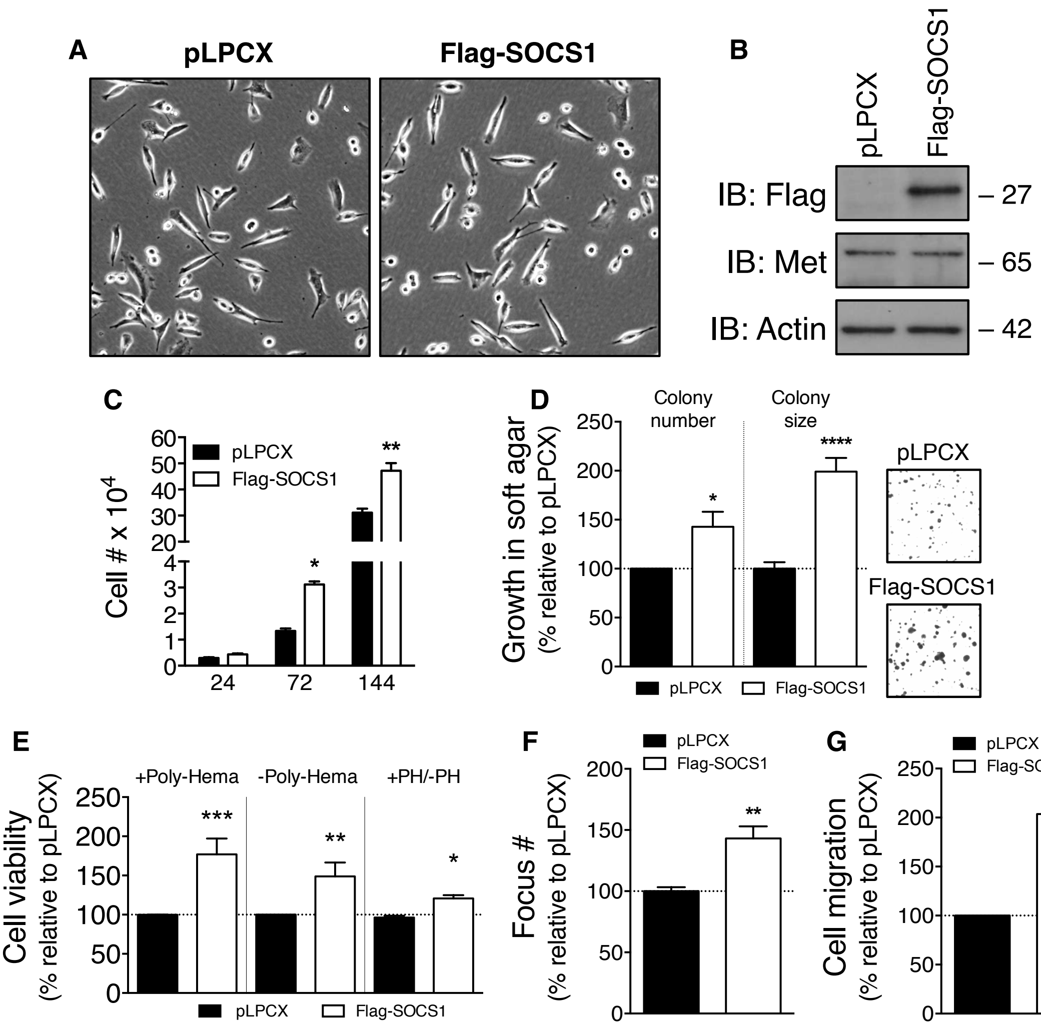

Supplemental Figure 2. SOCS1 promotes pro-oncogenic functions in intestinal epithelial cells transformed by a Met-derived oncoprotein. Gain-of-function studies for SOCS1 were performed in Tpr-Met-transformed rat intestinal epithelial cells (Tpr-Met-IEC6). Stable cell populations transduced with Flag-SOCS1 and the pLPCX empty vector were generated by retroviral infection. The impact of SOCS1 overexpression on various oncogenic features of the Tpr-Met-IEC6 cells was then evaluated in cell-based assays. (**A**) Morphology of Tpr-Met-IEC6 cells is not affected by the expression of Flag-SOCS1. (**B**) Flag-SOCS1 expression in Tpr-Met-IEC6 cells does not affect Tpr-Met protein levels. Flag-SOCS1 and Tpr-Met protein levels were evaluated by IB analysis. Cropped image of the blots are shown. (**C**) SOCS1 enhances cell growth in Tpr-Met-IEC6 cells. Cell-count assays were performed after seeding cells under adherent culture conditions in presence of serum. (**D**) SOCS1 in Tpr-Met-IEC6 cells enhances anchorage-independent growth in soft-agar. Photographs depict typical morphology of the colonies formed in soft agar by pLPCX and Flag-SOCS1 Tpr-Met-IEC6 cells. (**E**) SOCS1 in Tpr-Met-IEC6 cells promotes resistance to cell death induced by growth factors-deprivation and anoikis. Viability of the indicated cell populations was estimated 18 hours after their seeding in suspension (non-adhesive Poly-HEMA-coated plates) or adherent conditions. (**F**) SOCS1 in Tpr-Met-IEC6 cells enhances their capacity to bypass contact inhibition. The ability of the indicated cells to growth beyond confluence was determined in focus formation assays. (**G**) SOCS1 in Tpr-Met-IEC6 cells promoted migration. Migration capacity of the indicated cell populations was assessed in Boyden chamber assays. Cell populations were seeded in DMEM supplemented with 0.1% BSA onto 8.0μm polycarbonate membrane (Transwell®, LifeSciences) pre-coated with collagen type I (6ug/cm^2). DMEM supplemented with 10% FBS in the lower chamber was used as the chemoattractant. Following an incubation at 37°C/5% CO_2 for 3 hours, non-migrating cells were removed from the top membrane and migrating cells were counted following crystal violet staining. Number of migrating cells was normalized to that of proliferation over that 3-hour period. At least 2 independent experiments were performed in triplicate for each assay. The mean ± s.e.m values are expressed in percentage for Flag-SOCS1 Tpr-Met-IEC6 cells relative to that of the control pLPCX cells.
